# Bone targeting compounds for radiotherapy and imaging: *Me(III)-DOTA conjugates of bisphosphonic acid, pamidronic acid and zoledronic acid

**DOI:** 10.1186/s41181-016-0017-1

**Published:** 2016-09-23

**Authors:** M. Meckel, R. Bergmann, M. Miederer, F. Roesch

**Affiliations:** 1 Institute of Nuclear Chemistry, Johannes-Gutenberg-University Mainz, Fritz-Strassmann-Weg 2, Mainz, 55128 Germany; 2 Helmholtz-Zentrum Dresden-Rossendorf, Dresden, Germany; 3 Clinic of Nuclear Medicine, University Medicine Mainz, Mainz, Germany

**Keywords:** Hydroxy bisphosphonates, PET, Theranostics, Bone metastases, ^68^Ga, ^177^Lu, DOTA, Zoledronic acid

## Abstract

**Background:**

Bisphosphonates have a high adsorption on calcified tissues and are commonly used in the treatment of bone disorder diseases. Conjugates of bisphosphonates with macrocyclic chelators open new possibilities in bone targeted radionuclide imaging and therapy. Subsequent to positron emission tomography (PET) examinations utilizing ^68^Ga-labelled analogues, endoradiotheraphy with ^177^Lu-labelled macrocyclic bisphosphonates may have a great potential in the treatment of painful skeletal metastases.

**Methods:**

Based on the established pharmaceuticals pamidronate and zoledronate two new DOTA-α-OH-bisphosphonates, DOTA^PAM^ and DOTA^ZOL^(MM1.MZ) were successfully synthesized. The ligands were labelled with the positron emitting nuclide ^68^Ga and the β^-^ emitting nuclide ^177^Lu and compared in *in vitro* studies and in *ex vivo* biodistribution studies together with small animal PET and single photon emission computed tomography (SPECT) studies against [^18^F]NaF and a known DOTA-α-H-bisphosphonate conjugate (BPAPD) in healthy Wistar rats.

**Results:**

The new DOTA-bisphosphonates can be labelled in high yield of 80 to 95 % in 15 min with post-processed ^68^Ga and >98 % with ^177^Lu. The tracers showed very low uptake in soft tissue, a fast renal clearance and a high accumulation on bone. The best compound was [^68^Ga]DOTA^ZOL^ (SUV _Femur_ = 5.4 ± 0.6) followed by [^18^F]NaF (SUV _Femur_ = 4.8 ± 0.2), [^68^Ga]DOTA^PAM^ (SUV _Femur_ = 4.5 ± 0.2) and [^68^Ga]BPAPD (SUV _Femur_ = 3.2 ± 0.3). [^177^Lu]DOTA^ZOL^ showed a similar distribution as the diagnostic ^68^Ga complex.

**Conclusion:**

The ^68^Ga labelled compounds showed a promising pharmacokinetics, with similar uptake profile and distribution kinetics. Bone accumulation was highest for [^68^Ga]DOTA^ZOL^, which makes this compound probably an interesting bone targeting agent for a therapeutic approach with ^177^Lu. The therapeutic compound [^177^Lu]DOTA^ZOL^ showed a high target-to-background ratio. SPECT experiments showed concordance to the PET scans in healthy rats. [^68^Ga/^177^Lu]DOTA^ZOL^ appears to be a potential theranostic combination in the management of disseminated bone metastases.

**Electronic supplementary material:**

The online version of this article (doi:10.1186/s41181-016-0017-1) contains supplementary material, which is available to authorized users.

## Background

Radionuclide bone scintigraphy is usually applied to locate skeletal lesions from various cancer origins. The findings of the scintigraphy reflect the status of metabolic activity of the bone tissue as a result of tumour invasion. Positron emission tomography (PET) has one of the highest diagnostic sensitivity and specificity for the detection of skeletal metastases (Hamaoka et al. 2004), when used with the cyclotron produced [^18^F]NaF. Actually more common is the use of ^99m^Tc-bisphosphonate compounds in the whole body scintigraphy (Evan-Sapir et al. 2006). The positron emitter ^68^Ga has several advantages, which makes it an interesting object in the development of radiopharmaceuticals. First of all it is the cyclotron independent availability based on the ^68^Ge/^68^Ga generator system—similar to the availability of ^98m^Tc, and second, it is the intense emission of positrons—similar to ^18^F. Finally, it is the principle of the bifunctional chelating agent to design radiometal, in particular *Me(III), based pharmaceuticals. Substitution of β^+^ by β^-^ or α-particle emitters leads in principle to a theranostic concept, wherein diagnosis and therapy is combined in one precursor molecule. Since bisphosphonates (BP) show a high adsorption rate on bone, a number of new DOTA (1,4,7,10-tetraazacyclododecane-1,4,7,10-tetraacetic acid) based bone targeting molecules were described in the literature (Ogawa & Saji 2011), including conjugates of alendronic acid (Ogawa et al. 2009) (4-aminobutyl-1-hydroxy-1,1-bis[phosphonic acid]) and other α-H-bisphosphonates (Vitha et al. 2008). First promising human applications reported for [^68^Ga]BPAMD showed PET scans of equal quality to [^18^F]NaF and particularly a higher SUV_max_ in seleceted bone lesions (Fellner et al. 2010). In addition Passah et al. reported the use of a bisphosphonate conjugated to NOTA (1,4,7,10-tetraazacyclododecane-1,4,7,10-tetraacetic acid) chelator to detect bone metastases efficiently (Passah et al. 2014; Pfannkuchen et al. 2015).

**Chart 1:**
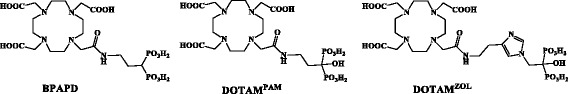
Structure of discussed DOTA-bisphosphonates

The evolution of modern osteoporosis drugs went from α-H bisphosphonates to α-OH and *N*-heteroaromatic bisphosphonates (Russell et al. 2008). Actually, the synthesis of DOTA-conjugated α-OH-bisphosphonates is more difficult compared to DOTA-conjugated α-H-bisphosphonates. But DOTA-α-OH-bisphosphonates may have higher skeletal retentions compared to DOTA-α-H-bisphosphonates. Consequently, we synthesized a pamidronate (3-aminopropyl-1-hydroxy-1,1-bis[phosphonic acid]) and a zoledronate derivative (([1-hydroxy-2-(1H-imidazol-1-yl)-ethylidene]bisphosphonic acid) as DOTA conjugates (Chart 1), labelled both with ^68^Ga and compared them in *in vivo* small animal PET and in *ex vivo* organ distribution studies in healthy Wistar rats with the known DOTA-α-H-bisphosphonate of similar structure (BPAPD) (for structures see Chart 1) and with [^18^F]NaF. Since DOTA offers the possibility of stable complexation of various radiometals, including isotopes relevant to therapy, the compounds discussed in this manuscript are potential precursors for a theranostic treatment of metabolic bone disorders, like skeletal metastases. Therefore, the best structure from the ^68^Ga experiments was further investigated in its ^177^Lu complex in organ distribution studies *ex vivo* and in small animal SPECT imaging studies *in vivo* in healthy Wistar rats.

## Results

### Synthesis

**Scheme 1:**
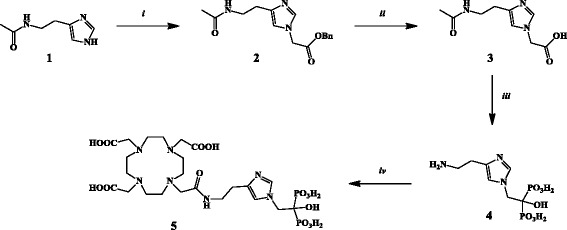
Synthesis of DOTA^ZOL^: (*i*) benzyl bromoacetate, DMF, Cs_2_CO_3_, 0 °C, 12 h. (*ii*) H_2_, Pd/C, MeOH, RT, 24 h. (*iii*) 1. PCl_3_ ,H_3_PO_3_, MeSO_3_H, 75 °C, 12 h, 2. H_2_O, 105 °C, 24 h. (*iv*) DOTA-NHS-ester, Na_2_CO_3_, 40 °C, 24 h

Synthesis of BPAPD is published in the literature (Vitha et al. 2008) as well as the synthesis of pamidronate (Kieczykowski et al. 1995). Compound (4) was prepared by a standard method for the synthesis of hydroxyl-bisphosphonates from carboxylic acids (Widler et al. 2002). The cleavage of the amide to form the primary amine was achieved in the same step, based on the strong acidic condition after hydrolysis of phosphorus trichloride. The imidazole derivative (3) was obtained from ω-*N*-acetylhistamine (1) after alkylation with benzyl bromoacetate and hydrogenation. Only the 1,4-(H-2 and H-5) imidazole (2) was formed during this reaction. A convenient method to discriminate between the 1,4- and the 1,5-alkylation product is described in the literature by the different coupling constants of the aromatic imidazole ring protons (Amino et al. 1989). (2) showed a coupling constant of *J*_2,5_ = 1.3 Hz, which is characteristic for the 1,4-product (range from 1.1 to 1.5 Hz). The final conjugation of the hydroxy-bisphosphonates (DOTA^PAM^ and DOTA^ZOL^) was performed *via* a modified route described by Ogawa et al. (2009). The compounds were first purified by preparative HPLC workup, and the remaining DOTA impurities were removed by solid phase extraction (SPE) using a weak anion exchanger resin (Scheme 1).

### Radiolabelling with n.c.a. ^68^Ga and n.c.a. ^177^Lu as well as quality control

Labelling with n.c.a. ^68^Ga was performed in sodium acetate buffer under heating (95 °C) on a thermo shaker for 15 min. Radiochemical yields (RCY) of the ^68^Ga-DOTA-bisphosphonate complexes were in the range between 80 and 95 %. After SPE using again a weak anion exchanger, the labelled compounds were present in 2 mL phosphate buffered saline (PBS) with a purity of over 98 %, ready for injection. Yields and purities were measured by radio thin layer chromatography (TLC) and HPLC (Additional file 1: Figure S1). For this HPLC quality controls aliquots of the purified reaction solutions were quenched with desferoxamine (DFO). Uncomplexed ^68^Ga remaining was instantly trapped by DFO to form [^68^Ga]DFO, which could be discriminated on RP-HPLC from the ^68^Ga-DOTA-BPs. This technique was not seen as a perfect HPLC method, since the radio labelled ^68^Ga-DOTA-bisphosphonates showed no retention on common RP-HPLC columns when using acetronitrile/water mixtures as solvents. Therefore, the radio HPLC method was further developed for the Lu-177 analogous using an ion pair reagent as running buffer. The method was later successfully adapted for the Ga-68 complexes (further details about radio-HPLC please refer to Additional file 1 section). Quantitative complexation of ^177^Lu(III) with DOTA^ZOL^ was obtained within 30 min at 98 °C. Radiochemicals yields were determined by radio-TLC and radio-HPLC.

### Adsorption experiments on apatite

**Fig. 1:**
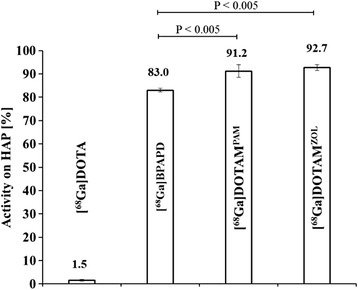
Adsorption of the three ^68^Ga-labelled DOTA-bisphosphonates on 20 mg HAP after 10 min incubation time, compared to [^68^Ga]DOTA shown in percent of the adsorbed fraction (N = 5)

Simple adsorption experiments on hydroxy apatite (HAP) proved the high accumulation of the ^68^Ga-labelled compounds on calcified surfaces like bone tissue, while [^68^Ga]DOTA as a control showed almost no adsorption. The hydroxy bisphosphonates [^68^Ga]DOTA^PAM^ (91.2 ± 2.7 %) and [^68^Ga]DOTA^ZOL^ (92.7 ± 1.3 %) showed an identical accumulation in this experiment (Fig. 1). Thus, binding of the pamidronate and zoledronate conjugates was superior compared to the α-H-bisphosphonate [^68^Ga]BPAPD (83.0 ± 0.8 %).

### *Ex vivo* biodistribution: ^68^Ga

The results of the *ex vivo* organ distribution in healthy Wistar rats of [^68^Ga]DOTA^PAM^, [^68^Ga]DOTA^ZOL^, [^68^Ga]BPAPD and [^18^F]NaF are presented in Tables 1 and 2, respectively. All ^68^Ga-labelled bisphosphonate compounds showed high accumulation in bone and low uptake in soft tissue. After 60 min the estimated activity in the skeleton was in the range of 40 to 50 % of the injected dose (ID) for [^68^Ga]BPAPD (38.8 ± 5.0 %ID), [^68^Ga]DOTA^PAM^ (48.4 ± 4.7 %ID) and [^68^Ga]DOTA^ZOL^ (42.7 ± 5.2 %ID), which is comparable to that of [^18^F]NaF (43.7 ± 1.9 %ID). Compared to the bisphosphonates, [^18^F]NaF showed a slight faster renal clearance and an overall lower concentration of activity in other organs, besides the bone. The bone-to-blood ratio determined from biodistribution were 3.7 for [^68^Ga]BPAPD, 7.6 for [^68^Ga]DOTA^PAM^, 11.5 for [^68^Ga]DOTA^ZOL^ and 97.4 for [^18^F]NaF.

**Table 1:** *Ex vivo* biodistribution of [^68^Ga]DOTA^PAM^, [^68^Ga]DOTA^ZOL^, [^68^Ga]BPAPD and [^18^F]NaF in healthy Wistar rats after 60 min p. i

Organ	[^68^Ga]DOTA^PAM^	[^68^Ga]DOTA^ZOL^	[^68^Ga]BPAPD^a^	[^18^F]NaF^a^
Lung	0.53 (0.16) ^b^	0.45 (0.11) ^b^	0.43 (0.08)	0.05 (0.01)
Liver	0.43 (0.04) ^b,d^	0.28 (0.03) ^b,d^	0.37 (0.11)	0.03 (0.01)
Spleen	0.31 (0.04) ^b,d^	0.17 (0.02) ^b,d^	0.23 (0.08)	0.13 (0.20)
Kidneys	0.48 (0.06) ^b^	0.53 (0.04) ^b^	0.56 (0.08)	0.11 (0.01)
Muscle	0.09 (0.02) ^c^	0.08 (0.02) ^c^	0.17 (0.02)	0.06 (0.05)
Heart	0.23 (0.02) ^b,c,d^	0.14 (0.04) ^b,c,d^	0.32 (0.09)	0.02 (0.01)
Blood	0.60 (0.03) ^b,c^	0.47 (0.19) ^b,c^	0.86 (0.21)	0.05 (0.01)
Intestine	0.28 (0.12) ^b^	0.14 (0.08) ^b^	0.26 (0.05)	0.02 (0.01)
Femur	4.53 (0.17) ^c,d^	5.40 (0.62) ^c,d^	3.21 (0.29)	4.87 (0.32)

Data are expressed in SUV. Each value represents the mean (S.D.) for five animals

^a^ Data taken from (Meckel et al. 2013)

^b^ *P* < 0.05 vs. [^18^F]NaF

^c^ *P* < 0.05 vs. [^68^Ga]BPAPD

^d^ *P* < 0.05 [^68^Ga]DOTA^PAM^ vs. [^68^Ga]DOTA^ZOL^

**Table 2:** Bone-to-blood ratios and calculated total activity in the skeleton in healthy Wistar rats, at 60 min p. i. of [^68^Ga]DOTA^PAM^, [^68^Ga]DOTA^ZOL^, [^68^Ga]BPAPD and [^18^F]NaF

compound	%ID in skeleton (S.D.)	bone to blood ratio
[^68^Ga]DOTA^PAM^	48.4 (4.7)	7.6
[^68^Ga]DOTA^ZOL^	42.7 (5.2)	11.5
[^68^Ga]BPAPD^a^	38.3 (5.0)	3.7
[^18^F]NaF^a^	43.7 (1.9)	97.4

Each value represents the mean for five animals

^a^Data taken from (Meckel et al. 2013)

### *In vivo* biodistribution: ^68^Ga

**Fig. 2:**
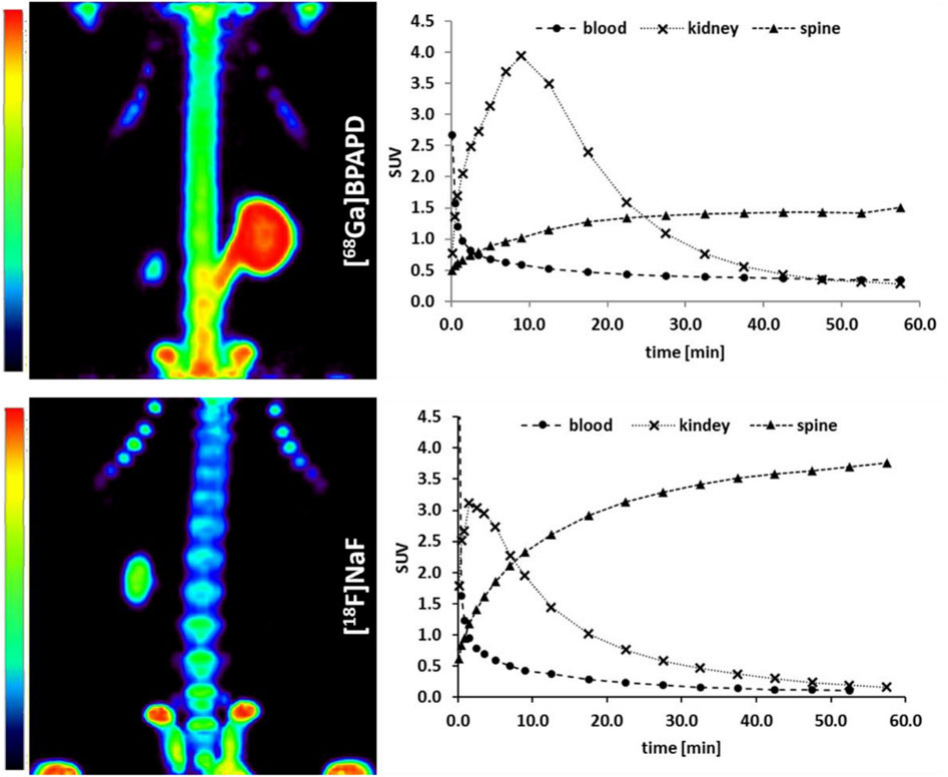
MIP (maximum intensity projection) in the thorax region of Wistar rats after 60 min p.i. and time activity curves (TAC) of [^68^Ga]BPAPD and [^18^F]NaF

**Fig. 3:**
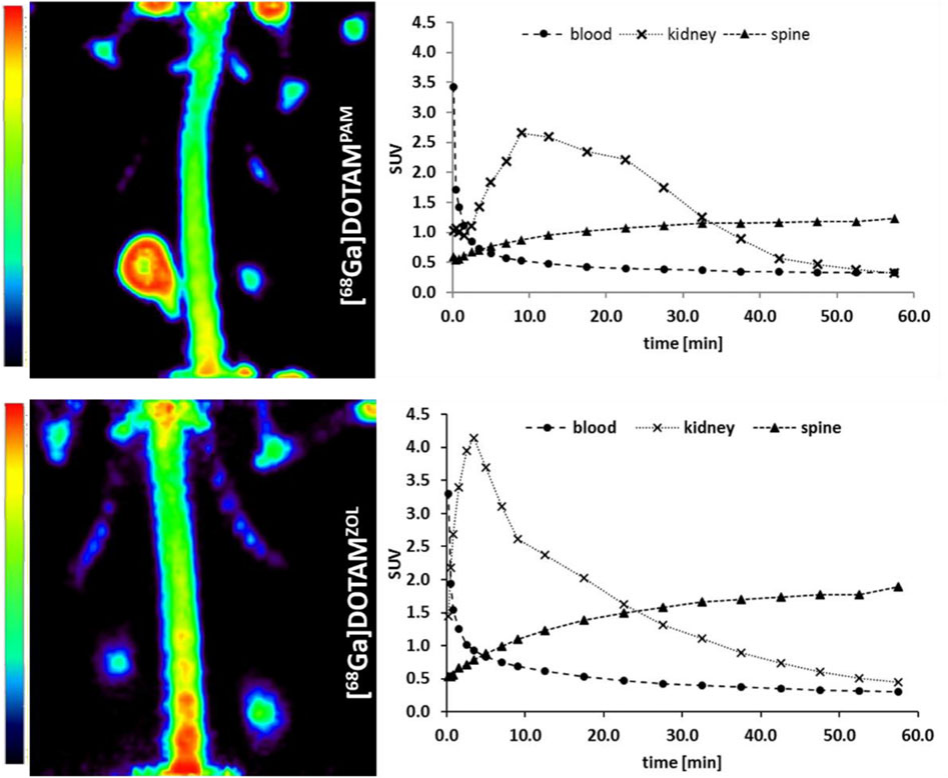
MIP (maximum intensity projection) in the thorax region of Wistar rats after 60 min p.i. and time activity curves (TAC) of [^68^Ga]DOTA^PAM^ and [^68^Ga]DOTA^ZOL^

Uptake kinetics and images from *in vivo* μPET studies are presented in Fig. 2. [^68^Ga]BPAPD, [^68^Ga]DOTA^PAM^ and [^68^Ga]DOTA^ZOL^ showed a rapid bone accumulation, reaching a plateau level at 45 min p. i. and a fast blood elimination. Distribution kinetics as time activity curves, Figs. 2 and 3, are very similar for all ^68^Ga-tracers. Variation in the kidney profile might be a result of differing response of the rats to anesthesia and diverging kidney activity. After 60 min all ^68^Ga-labelled bisphosphonates gave clearly visible bone scans. [^18^F]NaF PET showed in small animals a slight better quality and resolution.

### *Ex vivo* biodistribution: ^177^Lu

**Fig. 4:**
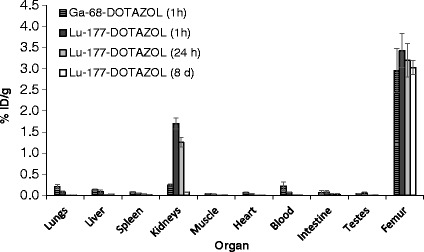
The graph represents the results from the *ex vivo* organ distribution of the ^68^Ga- and ^177^Lu-complexes of DOTA^ZOL^ in percent injected dose per gram

Because [^68^Ga]DOTA^ZOL^ appeared to be the compound with the highest bone accumulation among all the ^68^Ga-bisphosphonates studied in this manuscript, the ^177^Lu-analoque compound was investigated. The results of the organ distribution of [^177^Lu] DOTA^ZOL^ are represented in Table 3. The compound showed fast and high uptake in the skeleton and low accumulation in soft tissue. [^177^Lu]DOTA^ZOL^ showed the same skeletal retention as [^68^Ga]DOTA^ZOL^. Blood activity was lower for the ^177^Lu-complex, but renal clearance time showed to be longer. The activity in the skeleton was constant for 8 days, which clearly reveals the long biological half-life of [^177^Lu]DOTA^ZOL^ in the targeted tissue (Fig. 4). The complex was found to be intact in blood serum and urine samples over 1 h (radio-HPLC in Additional file 1: Figure S3).

**Table 3:** *Ex vivo* biodistribution of [^177^Lu]DOTA^ZOL^ in healthy Wistar rats after 1 h, 1 and 8 days p.i. , expressed in terms of %ID per organ and per gram tissue, respectively

Organ	% ID per Organ			% ID per gram		
	1 h	24 h	8 d	1 h	24 h	8 d
Lung	0.06 (0.01)	0.01 (0.00)	0.00 (0.00)	0.08 (0.02)	0.01 (0.01)	0.00 (0.00)
Liver	0.40 (0.13)	0.21 (0.07)	0.24 (0.04)	0.09 (0.04)	0.02 (0.01)	0.03 (0.01)
Spleen	0.01 (0.00)	0.01 (0.00)	0.01 (0.00)	0.05 (0.01)	0.02 (0.02)	0.02 (0.01)
Kidneys	1.78 (0.13)	1.64 (0.20)	0.10 (0.02)	1.70 (0.13)	1.25 (0.11)	0.07 (0.01)
Muscle	0.04 (0.02)	0.00 (0.00)	0.00 (0.00)	0.02 (0.00)	0.00 (0.00)	0.00 (0.00)
Heart	0.02 (0.00)	0.00 (0.00)	0.00 (0.00)	0.04 (0.01)	0.00 (0.00)	0.00 (0.00)
Blood	0.56 (0.07)	0.00 (0.00)	0.00 (0.00)	0.07 (0.01)	0.00 (0.00)	0.00 (0.00)
Intestine	1.59 (0.13)	0.38 (0.25)	0.29 (0.21)	0.08 (0.03)	0.02 (0.02)	0.02 (0.02)
Femur	2.11 (0.22)	1.89 (0.11)	2.25 (0.11)	3.43(0.41)	3.20 (0.40)	3.03 (0.17)
Skeleton	44.62 (5.00)	44.79 (4.82)	44.78 (3.71)	-	-	-

Each value represents the mean (S.D.) for four animals

### *In vivo* evaluation: ^177^Lu

**Fig. 5:**
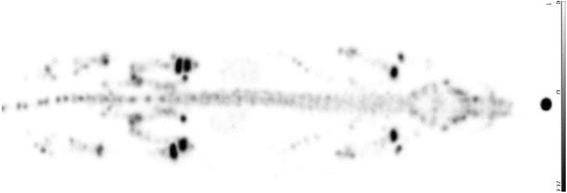
Whole body scintigraphy 1 h after administration of [^177^Lu]DOTA^ZOL^. The compound shows strong accumulation in the bone, and in particular in the high metabolic epiphyseal plates and other joint regions (the same effect was observed for the ^68^Ga-bisphosphonates)

SPECT image of [^177^Lu]DOTA^ZOL^ is exemplified in Fig. 5. The images show a good target to soft tissue ratio (49) with high skeletal accumulation (3.43 ± 0.40 %ID/g) and low background activity (blood: 0.07 ± 0.01 %ID/g; muscle: 0.02 ± 0.00 %ID/g).

## Discussion

The synthetic route for the DOTA-α-OH-bisphosphonate derivatives is distinctly different from that of the compound BPAPD. As a consequence, the α-H-bisphosphonate are definitely in a more easy way to synthesize. The coupling of the primary amine of the pamidronate or imidazole moiety in water via the *N*-hydroxysuccinimide ester of DOTA results in lower yields and the formation of problematic side products from ester hydrolysis, which have to be removed. Ogawa et al. (2009) separated the [^90^Y]DOTA-alendronate derivative from [^90^Y]DOTA by semipreparative HPLC. A combination of semipreparative HPLC and SPE showed the best purification results after the amide formation. Other groups reported the synthesis of protected alendronate, where the hydroxy bisphosphonate part is esterified by ethyl groups and a *tert-*butyldimethylsilyl ether (Vachal et al. 2006), leading to a new route for the developing of chelator α-OH- bisphosphonate conjugates (Suzuki et al. 2011). Nevertheless, this reaction seemed to be limited for compounds like alendronate. We were not able to prepare other derivatives with different alkyl or aromatic residues on the geminal hydroxy bisphosphonate by this synthetic pathway. The rearranged phosphonic acid derivative was the main product (Ruel et al. 1995; Brittelli 1985).

Complex formation with n.c.a. ^68^Ga was comparable for BPAPD, DOTA^PAM^ or DOTA^ZOL^ in terms of labelling yields and kinetics. Yields between 80 to 95 % were achieved after 15 min. Complex formation with n.c.a. ^177^Lu resulted in a quantitative radio-nuclide incorporation with DOTA^ZOL^ within 30 min reaction time.

Data from *in vitro* adsorption studies indicate a higher adsorption rate towards apatite for the DOTA-α-OH-bisphosphonate compared to DOTA-α-H-bisphosphonate, which could be further verified in *ex vivo* and in in vivo experiments. The DOTA pamidronate conjugate [^68^Ga]DOTA^PAM^ (SUV = 4.5 ± 0.2) showed a higher accumulation in bone compared to the DOTA bisphosphonate conjugate [^68^Ga]BPAPD (SUV = 3.2 ± 0.3). This proves the expectation that bisphosphonates containing an α-OH group have an enhanced accumulation on calcified surfaces, like it is discussed in the common literature. This leads to an almost doubled bone to blood ratio of 7.6 for [^68^Ga]DOTA^PAM^ compared to 3.7 for [^68^Ga]BPAPD. According to previous studies nitrogen-containing hydroxy bisphosphonates, especially *N*-heteroaromatic compounds showed an enhanced accumulation on bone. The *N*-heteroaromatic [^68^Ga]DOTA^ZOL^ showed the best bone to blood ratio of 11.5 and the best bone uptake (SUV = 5.4 ± 0.6) of all tested bisphosphonates. The overall skeleton accumulation is very similar for all tracers and it is comparable to that of [^18^F]NaF, which is in the range of 40 to 50 %ID.

In rats, all DOTA-based bisphosphonates showed PET images of good quality, which proves their potential as useful skeletal imaging agents. Basically, bisphosphonates are known to have a long biological half-life in bone. Since DOTA compounds are easy to label with β^-^ or α-particle emitters like ^177^Lu, ^213^Bi or ^225^Ac, the tracers discussed in this paper seemed to be excellently suitable for bone targeted radionuclide therapy. Therefore, we investigated the biodistribution of the best performed compound with its ^177^Lu analogous. [^177^Lu]DOTA^ZOL^ showed to have the same skeletal retention, but a faster blood clearance. The kidney uptake was found to be higher for the ^177^Lu-complex (1.78 ± 0.13 %ID/g) compared to the ^68^Ga-complex (0.24 ± 0.02 %ID/g). The results show that the incorporated radionuclide can have a significant influence on the organ distribution. The reason therefore can be explained by the different complexes, the six-dentate ^68^Ga-complex and the seven-dentate ^177^Lu-complex. The overall change of the Me(III)-DOTA-moiety may contribute to the pharmacology of the whole molecule. Nevertheless, the compound DOTA^ZOL^ maybe very well suited for the use of the theranostic pair ^68^Ga/^177^Lu to both detect and treat bone diseases which has to be further evaluated in dosimetry and tumor uptake studies.

It is also to mention that [^68^Ga]EDTMP was evaluated by Fellner et al. (2011) and Mitterhauser et al. (2007) as a PET imaging agent against [^18^F]NaF. They concluded that [^68^Ga]EDTMP has no advantage in image quality. In fact it was distinctly inferior and EDTMP is not suited as a bone targeting complexing agent for ^68^Ga^3+^ , in contrast to ^153^Sm^3+^ or ^177^Lu^3+^. Yousefnia et al. recently compared [^177^Lu]EDTMP against [^177^Lu]BPAMD (Yousefnia et al. 2016). His results substantiate the advantage of using macrocyclic conjugates of bisphosphonates. In contrast to the well-known bone targeting compounds [^18^F]NaF and [^177^Lu/^153^Sm]EDTMP DOTA-bisphosphonates can be used as a therapy and imaging agents, when labelled with β^-^ and positron emitting radio metals.

## Conclusion

In conclusion, α-OH and *N*-heteroaromatic bisphosphonates like DOTA^ZOL^ are more difficult to synthesize than simple α-H-bisphosphonates like BPAPD but show a significant improved accumulation in the skeleton. The new DOTA conjugated bisphosphonates allow for high radiochemical yields (80–95 %) with ^68^Ga after 15 min and a quantitative radiochemical yields with ^177^Lu after 30 min. Data from *in vivo* small animal PET/SPECT and *ex vivo* biodistribution showed fast blood clearance, low uptake in soft tissue and a high accumulation in the skeleton with [^68^Ga/^177^Lu]DOTA^ZOL^ being the best leading compound. In order to avoid problems with the nomenclature of this compound, in the following this molecule will be referred to also as [^68^Ga/^177^Lu]MM1.MZ.

## Methods

### General methods

Chemicals and solvents were commercially available in analytical, HPLC or grade and were purchased from Sigma-Aldrich (*Trace*SELECT®) or Merck KGaA. DOTA-NHS ester (1,4,7,10-Tetraazacyclododecane-1,4,7,10-tetraacetic acid mono(*N*-hydroxysuccinimidyl ester)) was synthesized according to a procedure described in the literature (Li et al. 2006) or purchased from Chematech (Dijon, France). Proton nuclear magnetic resonance (^1^H-NMR) spectra were recorded on a Bruker 300, ^31^P-NMR were recorded on a Bruker 600. Mass spectra were recorded on Agilent Technologies 6130 Quadrupole LC/MS spectrometer with ESI as ion source in positive or negative modes or on a Finnigan MAT-95 spectrometer with FD as ion source. TLC analyses were carried out with silica on aluminium foil (Merck). Radio-TLC analysis of labelled compounds were performed also with silica on aluminium foil (Merck) and a Canberra Packard Instant Imager. For HPLC and radio-HPLC a Waters-system 1525 with an UV- and a radio-detector (Berthold Technologies, Germany) was used. Radioactivity of samples were measured with an Aktivimeter Isomed 2010, MED (Nuklear-Medizintechnik Dresden GmbH). Radioactivity in tissue samples was determined by using a Wallac WIZARD2 automatic gamma counter (Perkin Elmer, Germany). N.c.a. ^177^Lu was provided by ITG (Garching, Germany) as LuCl_3_ in 0.05 M HCl. N.c.a. ^68^Ga was obtained from a ^68^Ge/^68^Ga generator system (Eckert & Ziegler). [^18^F]NaF was purchased from DKFZ (Deutsches Krebsforschungszentrum, Heidelberg, Germany).

### Synthesis of DOTA^PAM^

Pamidronate was synthesized according to a description by (Kieczykowski et al. 1995; Widler et al. 2002). 4.85 g (54.3 mmol) β-alanine was dissolved in 25 mL of methanesulfonic acid. 4.45 g (54.3 mmol) phosphorous acid was added and 10 mL (115 mmol) phosphorus trichloride was added slowly under vigorous stirring at 75 °C. The reaction mixture was kept under Argon atmosphere for 12 h at 75 °C. After cooling to room temperature, 50 g of crushed ice was added under vigorous stirring. The aqueous solution was heated for 12 h under reflux. After cooling to room temperature conc. aquaous NaOH was added until the formation of a white precipitate was observed. The mixture was stored in a fridge for 5 h to complete precipitation and filtered, washed with cold water and recrystallized from boiling water. Precipitation was initiated by adding ethanol. The white solid (5.3 g, 69 %) was filtered, washed with EtOH and dried in an oven at 60 °C. ^1^H-NMR (D_2_O/NaOD, 300 MHz): δ 2.30 (m, 2H), 3.34 (t, *J*_H_ = 6.8 Hz, 2H). ^31^P-NMR (D_2_O/NaOD, 162.05 MHz): δ 17.7 (s, 2P). ESI-MS(-) *m/z*: calculated 234.07 found 234.10 [M-H]^+^, ESI-MS(+) *m/z*: calculated 235.07; found 258.0 [M + Na]^+^.

DOTA^BP^ was synthesized according to a describtion by Ogawa et al. (2009). Twenty five (25) mg (0.11 mmol) pamidronate was dissolved in 1 mL 0.1 M Na_2_CO_3_ in water. 38 mg (0.05 mmol) DOTA-NHS-ester was dissolved in 0.5 mL deionized water and added to the pamidronate solution. The reaction solution was kept at 40 °C on a thermo shaker for 24 h. The pH was checked periodically and small amounts of aqueous Na_2_CO_3_ were used to keep the pH between 8 and 9. The crude mixture was purified initially by preparative HPLC (Phenomenex Synergy Hydro-RP 80, 10 μ, 250 × 30 mm, H_2_O + 0.1 % TFA). In the second step the crude product was purified further to remove DOTA impurities by solid phase extraction (SPE). An aqueous solution of the compound was passed over a silica NH_2_-phase (Merck LiChroprep NH_2_). After washing with water/methanol/water the product was eluted with H_2_O +2 % TFA. After lyophilisation 4.5 mg (14.5 %) of a white solid was obtained. ^1^H-NMR (D_2_O/NaOD, 300 MHz): δ 2.0–2.3 (b, 8H, cyclen-*CH*_*2*_), 2.0–2.33 (b, 2H,-*CH*_*2*_-), 2.9–3.5 (b, 8H, cyclen-*CH*_*2*_), 2.9–3.5 (b, 2H,-*CH*_*2*_-), 3.74 (bs, 8H, -*CH*_*2*_-CO). ^31^P-NMR (D_2_O/NaOD, 162.05 MHz): δ 18.3 (s, 2P). ESI-MS(+) *m/z*: calculated 621.18 found 1243.3 [2 M + H]^+^, 622.1 [M + H^+^], 311.6 [M + 2H]^2+^.

### Synthesis of DOTA^ZOL^

#### 1-(benzyl acetate) 4-(ethyl acetamide)-imidazole (2)

One (1) g (6.53 mmol) ω-*N*-acetylhistamine (1) was dissolved in 50 mL dry DMF. 4.4 g (13 mmol) ceasium carbonate was added. The solution was stirred under argon atmosphere and ice cooling. Benzyl bromoacetate (2.2 g; 13 mmol) was dissolved in 50 mL dry DMF and added slowly to the imidazole solution. The mixture was allowed to warm to room temperature and stirred for 12 h. Charcoal was added and the solids were filtered off. After removing the solvents under reduced pressure, the crude red residue was recrystallized from ethyl acetate to obtain 1.14 g (58 %) pale yellow crystals. ^1^H-NMR (CDCl_3_, 300 MHz): δ 1.97 (s, 3H, *CH*_*3*_-CO), 2.76 (t, *J*_H_ = 6.3 Hz, 2H, *CH*_*2*_-CH_2_), 3.53 (q, *J*_H_ = 6.0 Hz, 2H, *CH*_*2*_-CH_2_), 4.70 (s, 2H, N-*CH*_*2*_-CO), 5.22 (s, 2H, Bn-*CH*_*2*_-CO), 6.53 (bs, 1H, N*H*), 6.75 (d, *J*_H_ = 1.3 Hz, imidazole-*H*), 7.39 (m, 5H, benzyl), 7.44 (d, *J*_H_ = 1.3 Hz, 1H, imidazole-*H*). ). ESI-MS(+) *m/z*: calculated 301.14 found 302.3 [M + H]^+^, 603.2 [2 M + H]^+^.

#### 1-(1-hydroxy-ethane-1,1-bis(phosphonic acid)) 4-(ethyl amine)-imidazole (4)

Three hundred (300) mg (1 mmol) of (2) was dissolved in 20 mL of dry MeOH. Pd/C (10%w) was added and stirred under hydrogen atmosphere (5 bar) for 12 h. After removing the solvents the hydrogenated product (3) was weighted to determine yield (208 mg, 98 %). The acid (3) was used without further purification in the next step. One (1) mL methanesulfonic acid and 164 mg (2 eq.) phosphorous acid was added. The mixture was stirred at 75 °C and 300 mg (2.2 eq.) phosphorus trichloride was added slowly under argon atmosphere. After 12 h the reaction mixture was cooled down to room temperature and 2 mL of iced water was added. The aqueous solution was heated for 24 h under reflux. Charcoal was added and the solution was filtered. After cooling to room temperature concentrated aquaous NaOH was added until the formation of a white precipitate was observed. The mixture was stored in a fridge overnight to complete precipitation and filtered, washed with cold water and recrystallized from boiling water to obtain 88.6 mg (28 %) of a white solid. ^1^H-NMR (D_2_O/NaOD, 300 MHz): δ 2.46 (m, 2H, *CH*_*2*_-CH_2_), 2.66 (m, 2H, *CH*_*2*_-CH_2_), 4.28 (m, 2H, N-*CH*_*2*_-phosponate), 6.89 (s, 1H, imidazole-*H*), 7.54 (s, 1H, imidazole-*H*). ^31^P-NMR (D_2_O/NaOD, 162.05 MHz): δ 14.4. ESI-MS(+) *m/z*: calculated 315.04 found 316.05 [M + H]^+^, 338.04 [M + Na]^+^

#### DOTA^ZOL^ (5)

To 15.75 mg (0.05 mmol) (4) in 1 mL water, triethylamine (TEA) was added drop wise until all solids were dissolved. Thirty-eight (38) mg (0.05 mmol) DOTA-NHS-ester was dissolved in 0.5 mL water and added to the bisphosphonate solution (Ogawa et al. 2009). The reaction solution was kept at 50 °C on a thermo shaker for 24 h. The pH was checked periodically and small amounts of aqueous TEA were used to keep the pH between 8 and 9. The crude mixture was purified initially by preparative HPLC (Phenomenex Synergy Hydro-RP 80, 10 μ, 250 × 30 mm, solvent: H_2_O + 0.1 % TFA). In the second step the crude product was purified further to remove DOTA impurities by solid phase extraction (SPE). An aqueous solution of the compound was passed over a silica NH_2_-phase (Merck LiChroprep NH_2_). After washing with water/methanol/water the product was eluted with H_2_O + 2 % TFA. After lyophilisation 5.6 mg (15.7 %) of a white solid was obtained. ^1^H-NMR (D_2_O/NaOD, 300 MHz): δ 2.42 (m, 2H, *CH*_*2*_-CH_2_), 2.61 (m, 2H, *CH*_*2*_-CH_2_), 2.9–3.5 (b, 16H, cyclen-*CH*_*2*_), 3.75 (bs, 8H, -*CH*_*2*_-CO), 4.55 (m, 2H, N-*CH*_*2*_-phosponate), 7.28 (s, 1H, imidazole-*H*), 8.54 (s, 1H, imidazole-*H*). ^31^P-NMR (D_2_O/NaOD, 162.05 MHz): δ 14.3. ESI-MS(+) *m/z*: calculated 701.2 found 702.5 [M + H]^+^, 351.1 [M + 2H]^2+^.

#### Radiolabelling with n.c.a. ^68^Ga, ^177^Lu and quality control

N.c.a. ^68^Ga was obtained in 400 μL acetone/HCl by cationic post processing from a ^68^Ge/^68^Ga generator system eluted with 0.1 M HCl (Brittelli 1985; Zhernosekov et al. 2007). The labelling itself was performed by adding 500 μL sodium acetate buffer (0.5 M, pH = 4) and 25 nmol ligand to the ^68^Ga containing solution. The mixture was heated at 98 °C under moderate shaking on a thermo shaker for 15 min. Subsequently the solution was diluted to 5 mL with water and passed over a weak anion exchanger cartridge (25 mg, Merck LiChroprep NH_2_). After washing with 1 mL water the purified product was eluted with 2 mL PBS. Radiochemical yields and purities were determined by radio-TLC (Merck silica, solvent: acetone/acetylacetone/HCl, 10/10/1) and radio-HPLC (Merck Chromolite RP-18e 100 × 4.6 mm, gradient: H_2_O to acetonitrile). To discriminate between free ^68^Ga and ^68^Ga-bisphosphonates a small aliquot of the product solution was incubated for 1 min with a 0.25 M DFO (Desferoxamine) solution. DFO complexes unlabelled ^68^Ga instantly and shows a longer retention time on RP-18 HPLC (see Additional file 1: Figure S1). 1 GBq n.c.a. ^177^LuCl_3_ present in 500 μL of a 0.04 M HCl (obtained from ITG, Germany) was added to 20 nmol of the ligand DOTA^ZOL^ in 1 mL of a 0.25 M sodium acetate solution and heated for 30 min at 98 °C. Radiochemical yields were determined by radio-TLC using sodium citrate (0.1 M, pH = 4) as the solvent.

#### Adsorption experiments on apatite

Hydroxy apatite (HAP) (20 mg, Sigma-Aldrich, reagent grade powder) was incubated in isotonic saline (1 mL) for 24 h. Fifty (50) μL of the ^68^Ga-labelled bisphosphonate solution was added to the HAP suspension. After vortexing for 10 s, the suspension was incubated for 10 min at room temperature under moderate shaking on a thermo shaker. The supernatant was removed by centrifugation. The HAP fraction was washed with saline (0.5 mL) and the ^68^Ga radioactivity in the combined liquids and that of the HAP fraction were measured. ^68^Ga-complex binding to HAP was determined as percentage of ^68^Ga absorbed to HAP (*N* = 5) (Fellner et al. 2011).

#### Animal studies

Male Wistar rats were purchased from Charles River Laboratories Inc weighting 140–220 g. The animal experiments were carried out in consensus to institutional guidelines, the German welfare regulations and the *European Convention for the Protection of Vertebrate Animals for Experimental and other Scientific Purposes.*

#### Small animal PET and SPECT

Small animal PET and SPECT imaging was performed under general anesthesia with isoflurane inhalation. The rats were positioned supine in a Siemens Focus 120 μPET (Mediso nanoScan SPECT)and 15 to 18 MBq of the radio tracers (100 MBq for ^177^Lu-SPECT) were administrated in 0.5 mL isotonic saline intravenously (i.v.) via the tail vein. Images were reconstructed to OSEM 2D and files were processed using PMOD software (TAC) and Amine software (MIP). SPECT images were reconstructed using ROVER software.

#### Biodistribution

For each compound five animals were injected with 8 to 10 MBq of the radio tracers in 0.5 mL PBS/isotonic saline via the tail vein. The rats were sacrificed at 60 min and 1 d p.i. and organs of interest were excised, weighed and the radioactivity was measured decay corrected on a gamma counter. The amount of organ accumulation is expressed in SUV (standardised uptake value) by the equitation: SUV = (activity per g tissue)/(injected activity) x body weight. Activity in the skeleton was calculated as percentage of injected dose (%ID) by the following equitation: %ID(skeleton) = femur(%ID/g) x skeleton weight (g). The skeleton weight of the rats were calculated using: skeleton weight = 9.66 + 0.0355 x body weight (Vachal et al. 2006).

#### Statistical analysis

All data were expressed as mean ± SD. Groups were compared using the t-test. All statistical tests were two tailed, with a *P*-value of less than 0.05 representative for significance.

## Additional file

Additional file 1: **Figure S1.** Quality control of [^68^Ga]DOTA^ZOL^ by means of cross checked radio-HPLC and radio-TLC, as a representative of ^68^Ga-labelled bisphosphonates. A: After 15 min reaction time. B: After SPE purification. A(HPLC): R_t_([^68^Ga]DOTA^ZOL^) = 0.6 min. (88 %), R_t_([^68^Ga]DFO) = 4.1 min. (12 %). B(HPLC): R_t_([^68^Ga]DOTA^ZOL^) = 0.6 min. (98 %). A(TLC): R_f_([^68^Ga]DOTA^ZOL^) = 0.1 (85 %), R_f_([^68^Ga]acetylacetonate) = 0.9 (15 %). B(TLC): R_f_([^68^Ga]DOTA^ZOL^) = 0.1 (99 %). Control(TLC): R_f_([^68^Ga] acetylacetonate) = 0.9 (99 %). **Figure S2.** Radio-HPLC of [^177^Lu]DOTA^ZOL^ on a Zorbax 300SB-C18 9,4 × 250 mm 5 μ, A = 100 mM TEAP pH = 2,24, isocratic flow: 1 ml/min. **Figure S3.** Radio-HPLC of urine and blood samples after administration of [^177^Lu]DOTA^ZOL^. The HPLC method used for analysing the Lu-177 complexes is also suitable for the Ga-68 complexes of DOTA-Bisphosphonates. The Ga-68 complexes showed a different retention time based on the different character of the DOTA complex. This is also known for other DOTA-Compounds like DOTATOC or DOTATATE. **Figure S4.** Radio-HPLC of [^68^Ga]DOTA^ZOL^ on a Zorbax 300SB-C18 9,4 × 250 mm 5 μ, A = 100 mM TEAP pH = 2,24, isocratic flow: 1 ml/min. (DOCX 742 kb)
