# Supplementary material for: Bone targeting compounds for radiotherapy and imaging: *Me(III)-DOTA conjugates of bisphosphonic acid, pamidronic acid and zoledronic acid
Source: EJNMMI Radiopharm Chem. 2016 Sep 23;1:14. doi: 10.1186/s41181-016-0017-1 (PMC5843815; doi:10.1186/s41181-016-0017-1)
Supplement: Supplementary file 1 — Figure S1. Quality control of [68Ga]DOTAZOL by means of cross checked radio-HPLC and radio-TLC, as a representative of 68Ga-labelled bisphosphonates. A: After 15 min reaction time. B: After SPE purification. A(HPLC): Rt([68Ga]DOTAZOL) = 0.6 min. (88 %), Rt([68Ga]DFO) = 4.1 min. (12 %). B(HPLC): Rt([68Ga]DOTAZOL) = 0.6 min. (98 %). A(TLC): Rf([68Ga]DOTAZOL) = 0.1 (85 %), Rf([68Ga]acetylacetonate) = 0.9 (15 %). B(TLC): Rf([68Ga]DOTAZOL) = 0.1 (99 %). Control(TLC): Rf([68Ga] acetylacetonate) = 0.9 (99 %). Figure S2. Radio-HPLC of [177Lu]DOTAZOL on a Zorbax 300SB-C18 9,4 × 250 mm 5 μ, A = 100 mM TEAP pH = 2,24, isocratic flow: 1 ml/min. Figure S3. Radio-HPLC of urine and blood samples after administration of [177Lu]DOTAZOL. The HPLC method used for analysing the Lu-177 complexes is also suitable for the Ga-68 complexes of DOTA-Bisphosphonates. The Ga-68 complexes showed a different retention time based on the different character of the DOTA complex. This is also known for other DOTA-Compounds like DOTATOC or DOTATATE. Figure S4. Radio-HPLC of [68Ga]DOTAZOL on a Zorbax 300SB-C18 9,4 × 250 mm 5 μ, A = 100 mM TEAP pH = 2,24, isocratic flow: 1 ml/min. (DOCX 742 kb) [file 41181_2016_17_MOESM1_ESM.docx]

**Additional file 1**


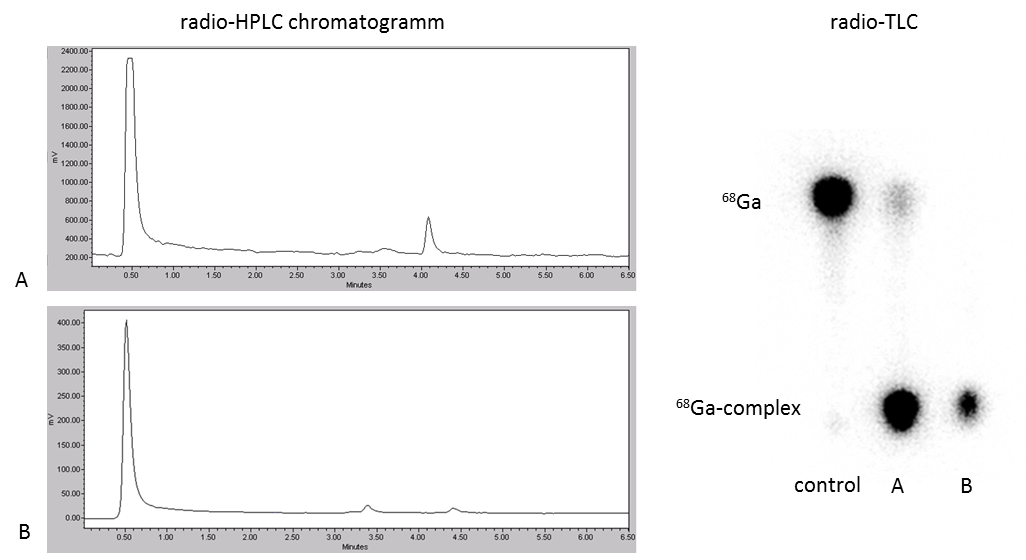


Figure S1. Quality control of [^68^Ga]DOTA^ZOL^ by means of cross checked radio-HPLC and radio-TLC, as a representative of ^68^Ga-labelled bisphosphonates. A: After 15 min reaction time. B: After SPE purification. A(HPLC): R_t_([^68^Ga]DOTA^ZOL^) =  0.6 min. (88%), R_t_([^68^Ga]DFO) =  4.1 min. (12%). B(HPLC): R_t_([^68^Ga]DOTA^ZOL^) =  0.6 min. (98%). A(TLC): R_f_([^68^Ga]DOTA^ZOL^) = 0.1 (85%), R_f_([^68^Ga]acetylacetonate) = 0.9 (15%). B(TLC): R_f_([^68^Ga]DOTA^ZOL^) = 0.1 (99%). Control(TLC): R_f_([^68^Ga] acetylacetonate) = 0.9 (99%).

Figure S2. Radio-HPLC of [^177^Lu]DOTA^ZOL^ on a Zorbax 300SB-C18 9,4 x 250mm 5µ, A = 100mM TEAP pH=2,24, isocratic flow: 1ml/min.

Figure S3. Radio-HPLC of urine and blood samples after administration of [^177^Lu]DOTA^ZOL^

The HPLC method used for analysing the Lu-177 complexes is also suitable for the Ga-68 complexes of DOTA-Bisphosphonates. The Ga-68 complexes showed a different retention time based on the different character of the DOTA complex. This is also known for other DOTA-Compounds like DOTATOC or DOTATATE.


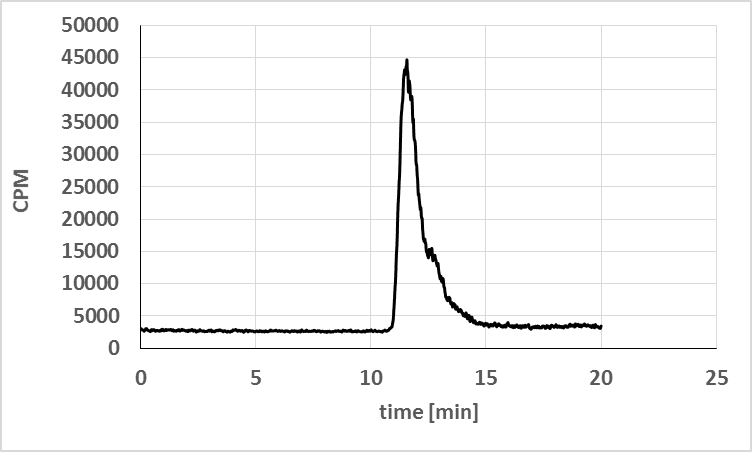


Figure S4. Radio-HPLC of [^68^Ga]DOTA^ZOL^ on a Zorbax 300SB-C18 9,4 x 250mm 5µ, A = 100mM TEAP pH=2,24, isocratic flow: 1ml/min.
